# Fracture Risk in Men and Women With Vertebral Fractures Identified Opportunistically on Routine Computed Tomography Scans and Not Treated for Osteoporosis: An Observational Cohort Study

**DOI:** 10.1002/jbm4.10736

**Published:** 2023-03-15

**Authors:** Michael Kriegbaum Skjødt, Joeri Nicolaes, Christopher Dyer Smith, Kim Rose Olsen, Cyrus Cooper, Cesar Libanati, Bo Abrahamsen

**Affiliations:** ^1^ Department of Medicine Holbæk Hospital Holbæk Denmark; ^2^ OPEN—Open Patient data Explorative Network, Department of Clinical Research University of Southern Denmark and Odense University Hospital Odense Denmark; ^3^ UCB Pharma Brussels Belgium; ^4^ Medical Image Computing, ESAT‐PSI, Department of Electrical Engineering KU Leuven Leuven Belgium; ^5^ DaCHE, Institute of Public Health University of Southern Denmark Odense Denmark; ^6^ MRC Lifecourse Epidemiology Center University of Southampton, Southampton General Hospital Southampton UK; ^7^ NDORMS, Nuffield Department of Orthopaedics, Rheumatology and Musculoskeletal Sciences Oxford University Hospitals Oxford UK

**Keywords:** FRACTURE PREVENTION, HEALTH SERVICES RESEARCH, OSTEOPOROSIS, RADIOLOGY, SCREENING

## Abstract

Vertebral fractures (VFs) have been associated with future fractures, yet few studies have evaluated whether this pertains to VFs available for identification on routine radiological imaging. We sought to evaluate the risk of subsequent fractures in subjects with VF identified opportunistically on computed tomography (CT) scans performed as part of routine clinical practice. From the radiology database of Holbæk Hospital we identified the first CT scan including the thorax and/or abdomen of 2000 consecutive men and women aged 50 years or older, performed from January 1, 2010 onward. The scans were assessed in a blinded approach to identify chest and lumbar VF, and these data linked to national Danish registers. Subjects were excluded if treated with an osteoporosis medication (OM) in the year prior to baseline (date of CT), and the remaining subjects with VF matched on age and sex in 1:2 ratio against subjects with no VF. We found that the risk of major osteoporotic fractures (hip, non‐cervical vertebral, humerus, and distal forearm fractures) was higher for subjects with VF than without VF: incidence rates (IRs) were 32.88 and 19.59 fractures per 1000 subject‐years, respectively, and the adjusted hazard ratio (HR_adj_) was 1.72 (95% confidence interval [CI], 1.03–2.86). Subsequent hip fracture IRs were 16.75 and 6.60; HR_adj_ 3.02 (95% CI, 1.39–6.55). There were no significant differences in other fracture outcomes (including a pooled estimate of any subsequent fracture, except face, skull, and fingers: IRs 41.52 and 31.38; HR_adj_ 1.31 [95% CI, 0.85–2.03]). Our findings suggest that subjects undergoing routine CT scans including the chest and/or abdomen are a high risk population in terms of fracture risk. Even within this group, subjects with VF are at higher risk of future major osteoporotic fracture (MOF), in particular hip fracture. Hence, systematic opportunistic screening for VF and subsequent fracture risk management is important to reduce the risk of new fractures. © 2023 The Authors. *JBMR Plus* published by Wiley Periodicals LLC on behalf of American Society for Bone and Mineral Research.

## Introduction

Osteoporosis is defined by detrimental changes to bone mass and microarchitecture, with a consequent increase in the susceptibility to fragility fractures.^(^ ^1^ ^)^ While such fractures can be associated with pain, disability, and mortality,^(^ ^2^, ^3^ ^)^ they also impose a large financial burden on the society. Across the European Union, the United Kingdom and Switzerland, an estimated total of 4.3 million fragility fractures occurred in 2019, while the estimated cost of fractures (including long‐term disability costs from fractures occurring before 2019) amounted to €55.3 billion.^(^ ^4^ ^)^

One frequently featured strategy to reduce individual‐level fracture risk—and as such the overall number of fragility fractures—is secondary fracture prevention.^(^ ^5^, ^6^ ^)^ In that regard, VFs are considered a hallmark of osteoporosis,^(^ ^7^ ^)^ predicting subsequent fractures,^(^ ^1^, ^8^ ^)^ and with this fracture risk being amenable to pharmaceutical intervention.^(^ ^9^ ^)^ However, although VFs are common in the aging population,^(^ ^10^ ^)^ less than one in three are clinically recognized at the time of occurrence,^(^ ^2^, ^11^, ^12^ ^)^ and systematic screening programs to capture undiagnosed VFs have not been widely implemented. Hence, to leverage the potential for secondary fracture prevention in individuals with VF, opportunistic identification and reporting of VFs from routine radiological imaging is encouraged.^(^ ^13^ ^)^ This should be considered a low‐hanging fruit, and would increase the utility of already undertaken radiological investigations and the associated radiation exposure.

Unfortunately, a number of studies have documented that a large proportion of patients with VF available on such imaging are not identified as fractured in radiology reports.^(^ ^14^, ^15^, ^16^, ^17^, ^18^, ^19^ ^)^ Suggested causes include—among others—a lack of routine examination of bone health when the imaging is performed for another indication,^(^ ^15^ ^)^ use of ambiguous terminology to describe the VF,^(^ ^15^, ^16^, ^20^ ^)^ and a lack of awareness about VFs among radiologists.^(^ ^19^ ^)^ In addition, although a number of studies have described an increased fracture risk in subjects with clinical VF as well as in subjects with VF identified purposively on baseline radiological imaging,^(^ ^21^, ^22^, ^23^, ^24^ ^)^ real‐world patient cohorts with opportunistically identified VF will likely differ substantially from the well‐defined cohorts of such observational studies. Few studies have investigated fracture risk in real‐world populations with opportunistically identified VF, and they are limited by assessing hip fracture risk only and not reporting relative risk estimates, respectively.^(^ ^14^, ^25^ ^)^ Hence it seems prudent to suggest that this lack of evidence plays a role in the aforementioned underreporting. To address this issue, the objective of this study is to evaluate the risk of subsequent fractures in subjects with VF identified opportunistically on CT scans performed as part of routine clinical practice.

## Subjects and Methods

This is an observational cohort study, established to evaluate the consequences of opportunistically identified VFs, when subjects are not treated with osteoporosis medications (OMs). We conducted blinded analysis of routine CT scans to identify prevalent chest and lumbar VFs, and subsequently established linkage to national Danish registers to enable formation of the analysis population as well as long‐term (up to 7 years) follow‐up. The reporting of this study follows the STROBE statement.^(^ ^26^ ^)^

The study was approved by the Danish Patient Safety Authority (3‐3013‐2687/1), Statistics Denmark (707480), and covered by the Danish Data Protection Agency approval for Region Zealand healthcare research (REG‐101‐2018). Ethics committee approval was not required.

### Patient and public involvement

The Patient and Relatives Council for Holbæk Hospital, which represents patients and their families, was consulted regarding study design.

### Setting

The CT scans were identified in the radiology database at Holbæk Hospital, Denmark, using scans performed from January 1, 2010 onward. Though a teaching hospital for the University of Copenhagen, Holbæk Hospital is located in a provincial area, and offers services entailing emergency medicine, internal medicine, orthopedic and abdominal surgery, among others.

Information from the CT scans was linked with national Danish registers on an individual level, made possible by the unique personal identification number assigned to all residents in Denmark.^(^ ^27^ ^)^ The registers are administrative in nature, and have been described in detail.^(^ ^27^, ^28^, ^29^, ^30^, ^31^ ^)^

From the Civil Registration System (CRS)^(^ ^28^ ^)^ we extracted information on sex, date of birth, migration in and out of Denmark, and country of origin.

From the National Patient Register (NPaR)^(^ ^29^ ^)^ we obtained information on public hospital contacts (both hospital admissions and outpatient visits) from 1994 onward, because at that time the register implemented the International Classification of Diseases 10th revision (ICD‐10). Private hospital contacts were added to the register in 2002. We extracted dates of admission and discharge, diagnosis codes, operation codes, procedure codes (eg, hospital administration of zoledronic acid) and dates, accident codes, and administrative information.

From the National Prescription Register (NPrR)^(^ ^30^ ^)^ we obtained information on prescriptions filled at Danish pharmacies from 1995 onward, including Anatomical Therapeutic Chemical classification (ATC) codes, dispensation date, and drug information.

From the Register of Causes of Death (RCD)^(^ ^31^ ^)^ we extracted the date of death.

No constraints were implemented for the look‐back period; ie, all data available from registry inclusion until the date of the CT scan (baseline) were used to inform the baseline characteristics. Subjects were followed for up to 7 years from baseline.

### Study population

The study included 2000 consecutive individuals with a CT scan including the chest and/or abdomen (other anatomic regions were allowed on the scan) performed from January 1, 2010 onward at Holbæk Hospital, Denmark, as part of routine clinical practice. Both men and women were included if aged 50 years or older at the time of the scan, and only the first eligible CT scan of each individual was included. In this context, *routine clinical practice* reflects that the scans were performed during—and as part of—day‐to‐day clinical work. There were no technical requirements to the CT images or protocol.

The CT scans were re‐evaluated in a two‐tiered process blinded to clinical information, in order to identify and grade prevalent chest and lumbar VFs. Initially, a medical doctor (CL) triaged the CT scans according to certain, potential, or no visible VF. The scans with *certain* or *potential* VF—together with a 5% subset of those scans deemed to have no VF—were then assessed by an external radiology service (Clario, Princeton, NJ, USA) for final vertebra‐level evaluation and grading of VFs. Each scan was read by a single, trained radiologist, according to the Genant Semiquantitative classification,^(^ ^32^ ^)^ adapted for evaluation of CT scans. A total of six radiologists participated in the diagnostic reading of the scans.

The fracture readings were subsequently linked with national registry data for formation of the analysis population and follow‐up. Subjects were excluded if no registry data was available or less than 1 year of registry data was available before baseline (defined as the date of the CT scan), emigrated before baseline, or treated with OM in the year prior to baseline. The remaining subjects with VF were matched on sex and age group (at the time of the CT scan, categorized into 5‐year age bands starting at age 50 years; ie, 50–54 years, 55–59 years, etc.) in a 1:2 ratio against subjects with no VF on the CT scan to form the analysis population.

In a scaling analysis, subjects with VF on the CT scan were matched on sex and age group (defined by birth year, categorized into 5‐year age bands; ie, 1905–1909, 1910–1914, etc.) against a general population cohort (*n* = 20,000) identified by Statistics Denmark among the population living in the same geographic region of Denmark. For this general population cohort, subjects were excluded prior to matching if conflicting registry data were present. Post‐matching, matched pairs were excluded if the comparator was aged less than 50 years or dead at the date of the CT scan of the matched case (baseline), less than 1 year of registry data was available prior to baseline, emigrated before baseline, or treated with OM in the year prior to baseline. Matched comparators were then randomly selected in a 3:1 ratio versus the VF cases.

### Outcomes

The primary outcome is the risk of any subsequent fracture—except face, skull, and fingers—after baseline in the VF on CT scan (exposed) cohort versus the no VF on CT scan (comparator) cohort. In planning the study, this fracture outcome was prioritized over major osteoporotic fracture (MOF) and hip fracture for reasons of study power, although the latter outcomes can be considered of larger clinical relevance.

The secondary outcomes are the risk of MOF and other fracture (defined as all non‐MOF, excluding face, skull, and fingers), respectively, in the VF on CT scan (exposed) cohort versus the no VF on CT scan (comparator) cohort. Major osteoporotic fracture (defined as hip, non‐cervical vertebral, humerus, and distal forearm fractures) were evaluated both as a composite endpoint and separately for each fracture location.

In the scaling analysis, outcomes similar to the primary and secondary outcomes described above were evaluated in the VF on CT scan (exposed) cohort versus the general population (comparator) cohort.

Fractures were identified in the NPaR, based on the occurrence of selected primary and secondary diagnosis codes (Table S1), including both inpatient and outpatient visits. To reduce the risk of erroneously counting a follow‐up for a preexisting fracture as new, fractures occurring within 6 months of a prior fracture at the same anatomical site were excluded. Similarly, no VFs were counted in the VF on CT scan cohort during the first 6 months after baseline, as 9 these could merely be follow‐up visits for the VF present on the baseline CT scan. A grace period of 6 months to avoid double‐counting of fractures has also been applied elsewhere.^(^ ^33^ ^)^ Fractures associated with accident codes indicating high‐energy accidents (Table S2) were excluded.

A more granular approach was applied in the identification of hip fractures. Hence, beyond a primary diagnosis code for hip fracture given for a hospitalization, patients had to have a relevant operation code with indication of the affected side (TUL‐code) during the same hospitalization (Table S1 for applied codes).

### Covariate identification and definition

Baseline sociodemographic characteristics and migration were identified in the CRS as described previously (please see the section *Setting*).

Baseline medical history was identified in the NPaR given the occurrence of at least one diagnosis code for disease entities defined a priori (Table S1). For a few diagnoses use of certain medications can be considered pathognomonic (eg, donepezil for dementia), and such ATC codes are included as disease identifiers.

Medication use was identified in the NPrR based on the filling of at least one relevant prescription at a Danish pharmacy. For OMs, we also identified hospital administration of denosumab or intravenous bisphosphonates by procedure codes (Table S1).

Charlson comorbidity index (CCI) score was calculated using the updated weights published by Quan and colleagues,^(^ ^34^ ^)^ applying no restrictions to the length of the look‐back period.

### Bias

We hypothesized a priori that the CT scan population would be somewhat selected and potentially at higher risk of subsequent fractures due to comorbid conditions, as compared to the general population—essentially reflecting a referral bias. Therefore, to also assess the utility of opportunistic identification of VF in a broader societal context, a scaling analysis was performed as described above (please see the sections *Study population and Outcomes*).

Nondifferential misclassification of the exposure is another source of potential bias, given that subjects in the no VF on CT scan (comparator) cohort could have a VF outside the CT field‐of‐view, and as such be erroneously classified as unfractured. If occurring, this would drive the hazard ratio of the risk of any subsequent fracture (primary outcome) toward 1. It is addressed in the sensitivity analyses, as described below (please see the section *Statistical analyses*).

### Sample size

The size of the study population (*N* = 2000) was determined to equal the expected number of relevant CT scans performed at Holbæk Hospital during 1 year. This was calculated to have >99% power to detect a 50% increase in subsequent fracture rate, given an expected VF prevalence of 26% at baseline (as observed in the control group of a similar study^(^ ^25^ ^)^), and a subsequent clinical fracture rate of 18% in 3 years—roughly translated to 60 fractures per 1000 patient‐years—in those with VF at baseline (as observed in the placebo group of the Fracture Intervention Trial [FIT] trial^(^ ^35^ ^)^). The sample size calculations demonstrated robustness against a smaller number of VF cases available for follow‐up, as well as a lower subsequent fracture rate.

### Statistical analyses

Baseline is considered the date of the index CT scan, and for the general population cohort the date of the CT scan of the matched case. Baseline characteristics are presented as median and interquartile range (IQR) for continuous covariates, while categorical covariates are given as counts and proportions. Between group differences are evaluated by the median test (Stata) and Pearsons chi‐squared (χ^2^), respectively.

The primary and secondary outcomes are evaluated by Cox proportional hazards regression models, censoring upon the first occurrence of a relevant fracture, initiation (first filled prescription or hospital‐administration) of OM, death, emigration, or at 7 years follow‐up, whichever occurred first. The proportional hazards assumption is evaluated by Schönfelds residuals, and for the primary outcome also visually by a log‐log plot. Fracture counts, follow‐up time, and incidence rates are assessed by survival analysis; ie, subjects contribute until censored. Mean follow‐up calculated as total follow‐up time divided by number of subjects. Number of subjects initiating treatment with an OM and number of subjects migrating out of Denmark are calculated from baseline until censored.

An adjusted model was developed for the risk of any subsequent fracture—except face, skull, and fingers—in the analysis population (primary outcome) and in the scaling analysis, respectively, using backward selection applying a statistical significance of *p* < 0.1 for confounder inclusion in the final model. Covariates evaluated for inclusion were any prior fracture (except face, skull, and fingers), and risk factors associated with osteoporosis and/or fractures (Table S1, Variable IDs 4.1–4.23.2, 5.3.2.1–5.3.2.2, and 6.1–6.7), while sex and age at baseline were forced into the models. For consistency, the adjusted models were applied unchanged in the evaluation of the secondary outcomes. Matching was lifted in the adjusted analyses for the program to run the model.

Preplanned subgroup analyses (definitions and findings listed in Table S3) were performed for the primary outcome. These include stratifications by age, sex, any malignancy within 3 years before baseline, known bone diseases (excluding osteoporosis), known osteoporosis, number of VF, worst VF, and position of VF. A subgroup analysis according to CCI score was added post hoc. Age was grouped into decennials while pooling the oldest old; ie. 50–59, 60–69, 70–79, and 80+ years. The highest number of VF (four or more) were pooled in one group, while the highest CCI score (three or higher) were also pooled in one group. Analyses were performed by the implementation of an interaction term evaluated by Wald test, except for age and sex for which separate analyses for each category were performed.

Sensitivity analyses were performed, exploring the robustness of the primary outcome to loss to follow‐up (occurring if a subject migrated out of Denmark before censored, in which case subsequent events would not be identifiable in the Danish registers), different exclusion and censoring conditions based on use of OMs, misclassification of the exposure, implementation of a competing risk of death, and an evaluation of the control of confounders (definitions and findings listed in Table S4). To address the risk of nondifferential misclassification of the exposure, a post hoc analysis was conducted including only those comparator subjects with all thoracic and lumbar vertebrae (T_1_–L_5_) visible on the CT scan, safeguarding against such misclassification (Table S4, “Misclassification bias 2”).

Given the nature of the study, with presence or absence of a variable defined by occurrence and non‐occurrence of a given code, respectively, evaluating missing data becomes nonsensical. There were no missing data for the baseline variables age, sex, and country of origin. Hence, missing data is not further addressed in the analyses. Analyses were not adjusted for multiplicity.

Analyses were performed using Stata version 16 and 17 (StataCorp, College Station, TX, USA).

## Results

### Formation of the analysis population

**Fig. 1:**
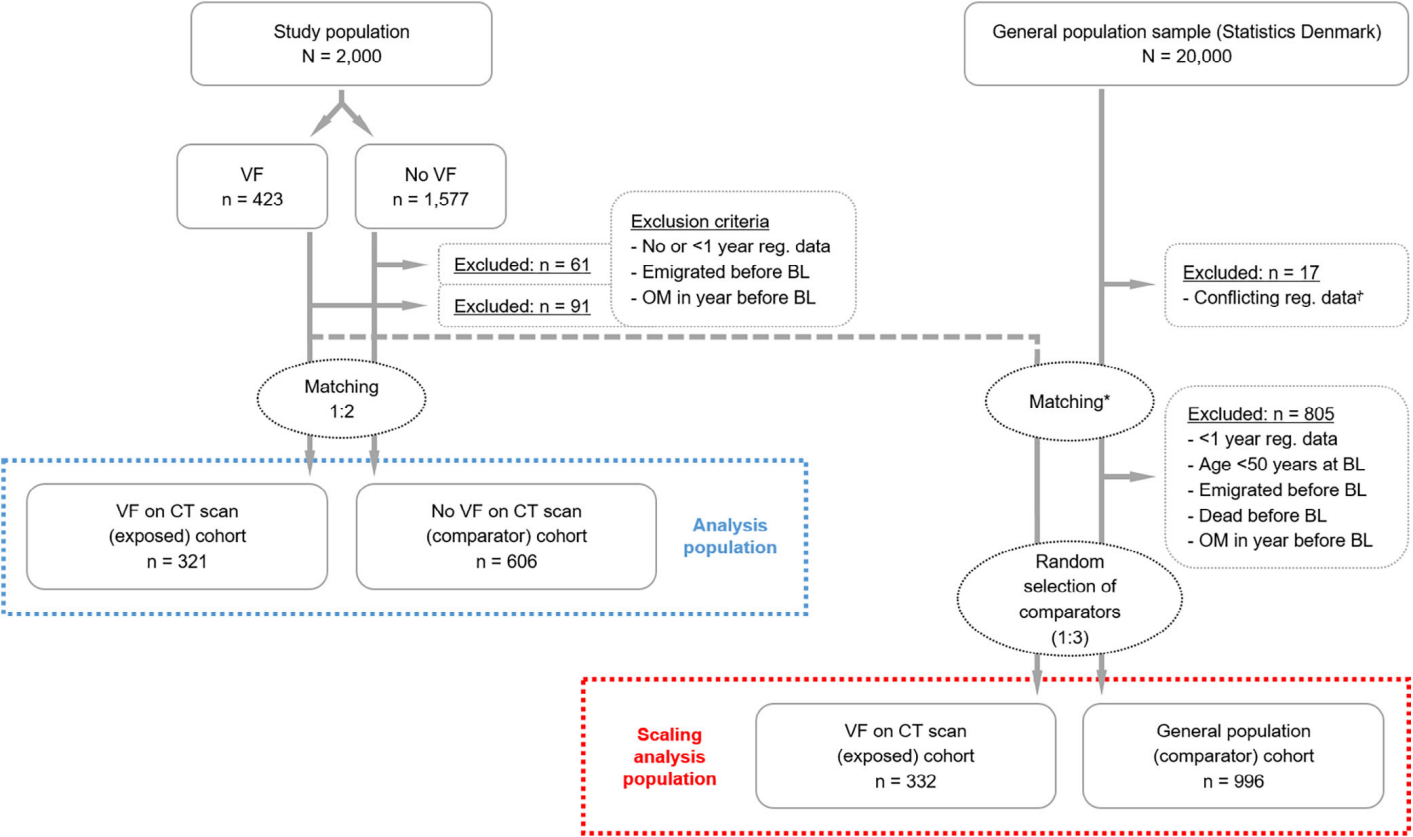
Flowchart. The left side of the figure shows the formation of the analysis population, and the right side of the figure shows the formation of the scaling analysis population. The number of subjects excluded has been pooled due to a small *n* in some of the subgroups. During the matching process, subjects with VF with no matched comparators were omitted from the analysis population, while subjects with only one matched comparator were retained. *Index date (date of case CT scan) transferred to general population comparators upon matching. ^†^Subjects excluded prior to matching due to conflicting registry records. BL = baseline; CT = computed tomography; OM = osteoporosis medication; reg. = registry; VF = vertebral fracture.

In the evaluation of the 2000 CT scans, 423 (21.2%) subjects had at least one VF on the CT scan, while 1577 subjects had no visible VF. From the VF on CT scan and the no VF on CT scan groups, a total of 91 (21.5%) and 61 (3.9%) subjects were excluded, respectively. After matching the remaining subjects, the VF (exposed) cohort was constituted of 321 subjects, and the no VF (comparator) cohort of 606 subjects. The flowchart is shown in Fig. 1.

### Findings from the CT scans

**Fig. 2:**
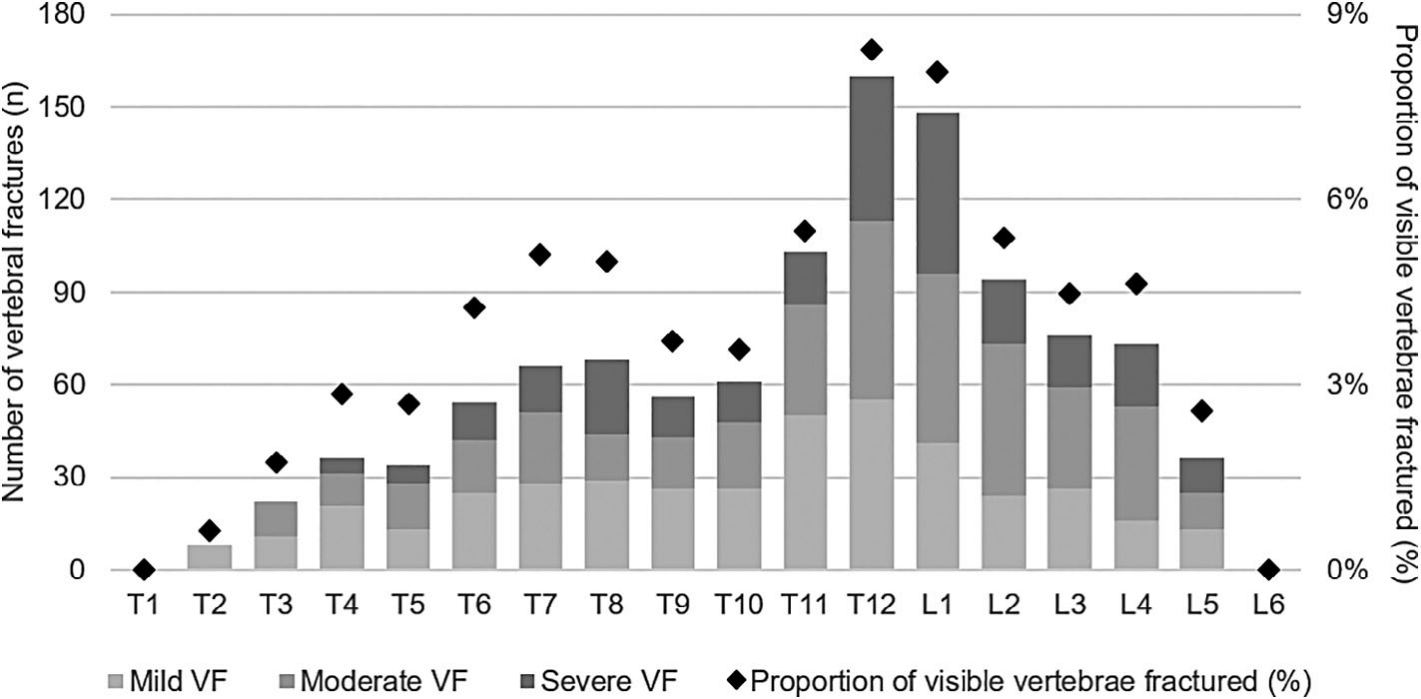
Findings from the CT scan evaluation. The figure demonstrates the number of mild, moderate, and severe VFs identified during the evaluation of the CT scans (*N* = 2000), stratified according to vertebral level. Also shown (diamonds) is the proportion of visible vertebrae deemed to be fractured. Fracture numbers (and proportions) not reported if *n* < 5 or if retained to maintain confidentiality. Presence of L_6_ documented in Clario readings only. CT = computed tomography; VF = vertebral fracture.

In the 2000 CT scans, a total of 1111 VFs were identified in 423 (21.2%) subjects. Of these, 691 (62.2%) VFs in 310 (73.3%) subjects were moderate or severe. The distribution of VFs stratified according to vertebral level is depicted in Fig. 2, showing the highest occurrence of VFs at T_12_–L_1_ nearing a prevalence of 9%. Other common fracture sites were T_7_–T_8_, T_11_, and L_2_–L_4_.

Within the VF cohort (*n* = 321), the worst VF identified in the evaluation of the CT scans was severe in 104 (32.4%) subjects, moderate in 117 (36.4%) subjects, and mild in the remaining 100 (31.2%) subjects. A single VF was identified in 143 (44.5%) subjects, two VFs in 81 (25.2%), three VFs in 39 (12.1%), and four or more VFs in 58 (18.1%) subjects.

### Baseline characteristics

Table 1 shows the baseline characteristics of the VF and the no VF cohorts. The majority of subjects were of Danish descent. There was no statistically significant difference in the median CCI score, yet the proportion of subjects with a baseline history of any fracture or MOF were higher in the VF cohort. Consistent with these findings, a larger proportion of the VF subjects had a diagnosis code for osteoporosis.

**Table 1:** Baseline Characteristics

Characteristic	VF on CT scan (exposed cohort)	No VF on CT scan (comparator cohort)	*p*
*N*	321	606	
Age (years), median (IQR)	73 (65–79)	73 (65–79)	0.75
Sex, men, *n* (%)	172 (53.6)	322 (53.1)	0.90
Country of origin, Denmark, *n* (%)	312 (97.2)	592 (97.7)	0.73
CCI score, median (IQR)	2 (0–3)	1 (0–2)	0.09
Any prior fracture, *n* (%)^a^	112 (34.9)	142 (23.4)	<0.001
Prior MOF	70 (21.8)	71 (11.7)	<0.001
Prior VF^b^	20 (6.2)	*n* < 5	N/A
Osteoporosis, *n* (%)	14 (4.4)	6 (1.0)	<0.001
Glucocorticoid therapy, *n* (%)^c^	53 (16.5)	78 (12.9)	0.13
Hormone replacement therapy, *n* (%)^c^	15 (4.7)	45 (7.4)	0.11
Malignancies, *n* (%)	127 (39.6)	219 (36.1)	0.30
Rheumatoid arthritis, *n* (%)	6 (1.9)	13 (2.1)	0.78
Type 1 diabetes mellitus, *n* (%)	18 (5.6)	38 (6.3)	0.69
Type 2 diabetes mellitus, *n* (%)	37 (11.5)	73 (12.0)	0.82

Abbreviations: CCI = Charlson Comorbidity Index; CT = computed tomography; IQR = interquartile range; MOF = major osteoporotic fracture; N/A = not available; VF = vertebral fracture.

^a^ Not including face, skull, or fingers.

^b^ Including cervical VF.

^c^ In the year prior to baseline.

### Primary outcome: Risk of any subsequent fracture

Subjects were followed for a mean of 2.3 and 3.4 years in the VF and no VF cohorts, respectively, giving a total follow‐up time of 2818 subject‐years. During follow‐up, 31 and 65 subjects experienced any first incident fracture (Table 2), yielding incidence rates (IRs) of 41.52 and 31.38 fractures per 1000 subject‐years in the VF and the no VF cohorts, respectively. Only a small subset of subjects, 29 (9%) in the VF cohort and 38 (6%) in the no VF cohort, were censored due to initiation of treatment with an OM during follow‐up. Few (*n* < 5) subjects across both cohorts were lost to follow‐up due to migration out of Denmark.

**Table 2:** Risk of Subsequent Fractures: Findings for the Primary and Secondary Outcomes

	Fracture counts *n* (%) subjects with first fracture		Incidence rate per 1000 subject‐years (95% CI)		Risk estimate hazard ratio (95% CI; *p*)	
Future fracture type	VF on CT scan (exposed cohort)	No VF on CT scan (comparator cohort)	VF on CT scan (exposed cohort)	No VF on CT scan (comparator cohort)	Crude	Adjusted^a^
Any	31 (9.7)	65 (10.7)	41.52 (29.20–59.04)	31.38 (24.61–40.02)	1.30 (0.85–2.00; *p* = 0.23)	1.31 (0.85–2.03; *p* = 0.23)
MOF	25 (7.8)	41 (6.8)	32.88 (22.22–48.66)	19.59 (14.42–26.60)	1.66 (1.00–2.74; *p* = 0.049)	1.72 (1.03–2.86; *p* = 0.04)
Other	14 (4.4)	35 (5.8)	18.24 (10.80–30.80)	16.64 (11.94–23.17)	1.07 (0.57–1.98; *p* = 0.84)	1.03 (0.55–1.93; *p* = 0.93)
Hip	13 (4.0)	14 (2.3)	16.75 (9.72–28.84)	6.60 (3.91–11.14)	2.70 (1.27–5.75; *p* = 0.01)	3.02 (1.39–6.55; *p* < 0.01)
Vertebral	6 (1.9)	6 (1.0)	7.63 (3.43–16.97)	2.82 (1.27–6.28)	3.06 (0.92–10.21; *p* = 0.07)	2.44 (0.77–7.76; *p* = 0.13)
Humerus^b^	16 (1.7)		5.52 (3.38–9.01)		0.82 (0.26–2.57; *p* = 0.73)	0.87 (0.28–2.73; *p* = 0.81)
Distal forearm^b^	20 (2.2)		6.91 (4.46–10.71)		0.63 (0.20–1.92; *p* = 0.41)	0.67 (0.22–2.07; *p* = 0.49)

*Note*: Fracture counts, incidence rates, and risk estimates for the primary outcome (risk of any subsequent fracture—except face, skull, and fingers—in the VF on CT scan [exposed] cohort versus the no VF on CT scan [comparator] cohort) and according to subsequent fracture location (secondary outcome).

Abbreviations: CI = confidence interval; CT = computed tomography; MOF = major osteoporotic fracture; VF = vertebral fracture.

^a^ Adjusted for sex, age, baseline presence of any prior fracture, anorexia, ever use of antidepressants, and ever use of proton pump inhibitors. Matching lifted for the adjusted analyses.

^b^ Numbers pooled across the cohorts as number of events <5 in one of the cohorts.

**Fig. 3:**
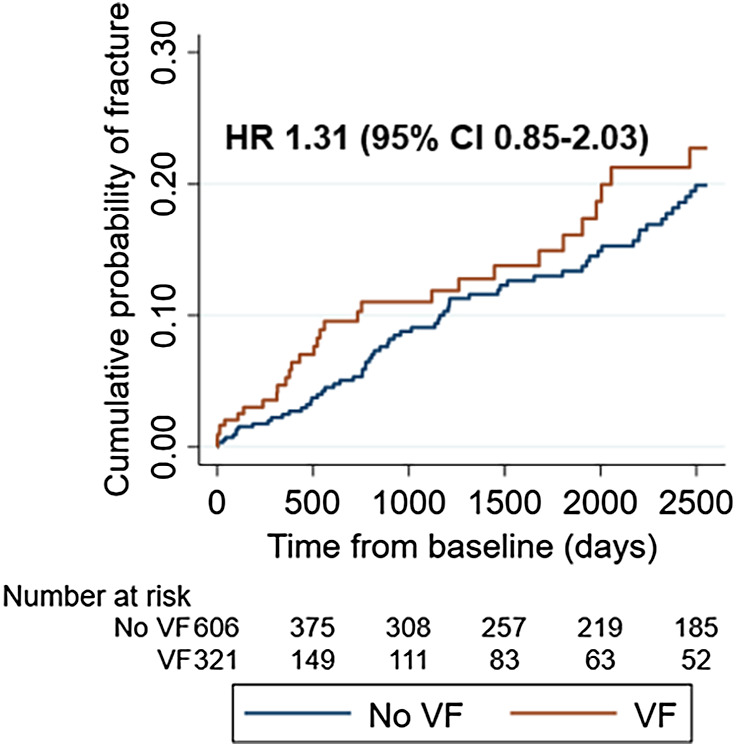
Cumulative probability of any subsequent fracture. This Kaplan‐Meier failure function demonstrates the cumulative probability of any subsequent fracture—except face, skull, and fingers—in the VF on CT scan (exposed) cohort versus the no VF on CT scan (comparator) cohort. Also shown is the hazard ratio for any subsequent fracture (except face, skull, and fingers), adjusted for age, sex, baseline presence of any prior fracture, anorexia, ever use of antidepressants, and ever use of proton pump inhibitors. CI = confidence interval; CT = computed tomography; HR = hazard ratio; VF = vertebral fracture.

The Kaplan‐Meier failure function for the risk of any subsequent fracture is shown in Fig. 3. The curves of the VF and the no VF cohorts separated early after baseline. Although the cumulative fracture probability remained higher in the VF cohort throughout the study, the difference between the curves fluctuated. The number of subjects at risk in the VF cohort is relatively small in the later part of the follow‐up period.

The crude hazard ratio (HR) was 1.30 for the risk of any subsequent fracture in the VF cohort versus the no VF cohort, with no change in the effect size in the multivariate model (HR 1.31, adjusted for sex, age, baseline presence of any prior fracture, anorexia, ever use of antidepressants, and ever use of proton pump inhibitors), with neither estimate being statistically significant (*p* = 0.23). The proportional hazards assumption was met (log‐log plot shown in Fig. S1).

Subgroup analyses did not identify any statistically significant interactions (Table S3). Nonetheless, there were indications of higher HRs for any subsequent fracture in the older age groups (*p* for interaction not available for this analysis). Importantly, there was a trend (*p* for interaction not significant) toward higher risk of any subsequent fracture with increasing severity of the worst VF at baseline: Adjusted HR (HR_adj_) 0.94 for subjects with mild VF, 1.14 for moderate VF, and 2.08 for severe VF, with the latter being statistically significant.

The primary outcome was unchanged across most sensitivity analyses (Table S4). In the analysis of the risk of misclassification of the exposure (Table S4, “Misclassification bias 2”), including only those comparator subjects with all thoracic and lumbar vertebrae (T_1_–L_5_) visible on the CT scan, the HR increased markedly (HR_adj_ 1.81 [95% CI, 0.86–3.81]), yet remained nonsignificant. Furthermore, the sensitivity analysis factoring in the competing risk of death demonstrated a reduction of the hazard ratio estimate (HR_adj_ 0.87 [95% CI, 0.56–1.36]), indicating a higher competing risk of death in the VF cohort versus the no VF cohort.

### Secondary outcomes: Risk of major osteoporotic fracture and other fracture

**Fig. 4:**
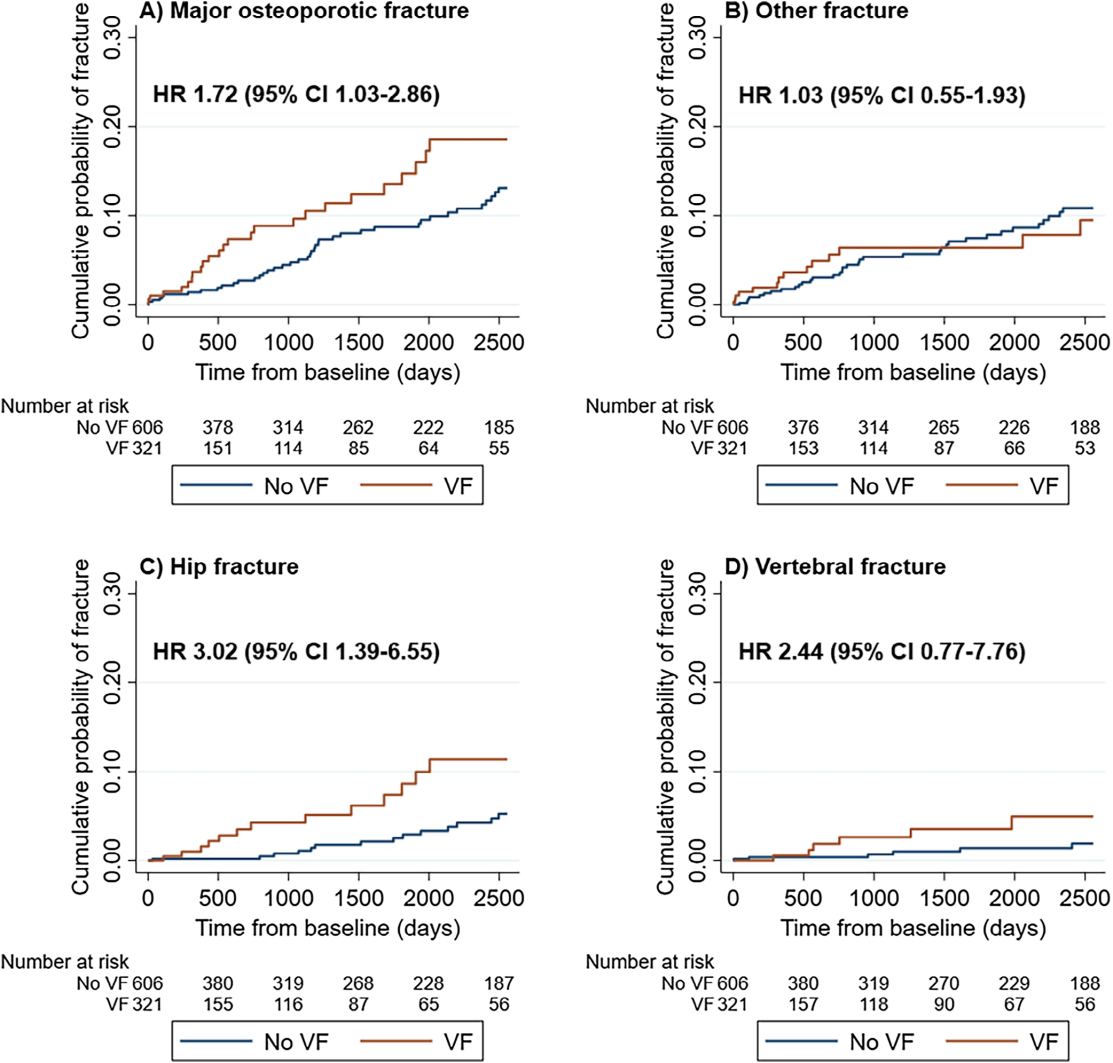
Cumulative probability of fracture according to subsequent fracture location. Panel A, major osteoporotic fracture (MOF); B, other fracture; C, hip fracture; D, vertebral fracture. The figure shows the Kaplan–Meier failure functions according to subsequent fracture location in the VF on CT scan (exposed) cohort vs the no VF on CT scan (comparator) cohort. On each panel is shown the hazard ratio (95% CI) for subsequent fracture according to subsequent fracture location, adjusted for age, sex, baseline presence of any prior fracture, anorexia, ever use of antidepressants, and ever use of proton pump inhibitors. Panels for humerus fracture and distal forearm fracture not shown as number of events <5 in one of the cohorts. CI, confidence interval; HR, hazard ratio; MOF, major osteoporotic fracture; VF, vertebral fracture.

There was a significantly increased risk of MOF (HR_adj_ 1.72 [95% CI, 1.03–2.86; *p* = 0.04]) in the VF cohort versus the no VF cohort (Fig. 4 and Table 2). When evaluating the components of MOF separately, the excess risk was most pronounced for hip fracture (HR_adj_ 3.02 [95% CI, 1.39–6.55; *p* < 0.01]), while a nonsignificant, numerically higher risk of subsequent VF was also observed (HR_adj_ 2.44 [95% CI, 0.77–7.76; *p* = 0.13]).

We found no differences between the cohorts in the risk of other fracture (HR_adj_ 1.03 [95% CI, 0.55–1.93; *p* = 0.93]), humerus fracture (HR_adj_ 0.87 [95% CI, 0.28–2.73; *p* = 0.81]), nor distal forearm fracture (HR_adj_ 0.67 [95% CI, 0.22–2.07; *p* = 0.49]).

### Fracture risk compared with the general population (scaling analysis)

In this pre‐planned analysis, the group with VF on the CT scan was matched against a general population cohort to scale risk estimates against the general population. The flowchart is shown in Fig. 1, and 332 and 996 subjects were matched into the VF on CT scan cohort and the general population cohort, respectively. Baseline characteristics (Table S5) showed that the sex‐ and age‐distributions were similar at baseline, yet the VF cohort appeared markedly more ill at baseline, manifesting in a significantly higher CCI‐score (median CCI‐score 2 versus 0, *p* < 0.001). The mean follow‐up time from baseline to any first incident fracture was 2.3 years in the VF cohort and 5.3 years in the general population cohort. We found that the absolute fracture risk was substantially lower in the general population cohort than in the no VF on CT scan cohort. As such, incidence rates were higher in the no VF cohort (Table 2) for any fracture (+44%), MOF (+38%), and other fracture (+67%) as compared to the general population cohort (Table S6), indicating a high absolute risk of fracture in subjects undergoing CT scans, even in the absence of prevalent VF.

There was a statistically significant 60% increase (HR_adj_ 1.60 [95% CI, 1.07–2.40; *p* = 0.02]) in the risk of any subsequent fracture (except face, skull, and fingers) in the VF cohort versus the general population cohort (Table S6 and Figs. S2 and S3). Similarly, significant increases in the risk of hip fracture (HR_adj_ 2.55 [95% CI, 1.35–4.81; *p* < 0.01]) and VF (HR_adj_ 4.66 [95% CI, 1.51–14.36; *p* < 0.01]) were observed, driving an increased risk of MOF (HR_adj_ 2.04 [95% CI, 1.29–3.23; *p* < 0.01]). There were no significant differences in the risk of other fracture, humerus fracture, nor distal forearm fracture.

Schönfelds residuals were consistent with the proportional hazards assumption (ie, nonsignificant for all analyses [data not shown]; not evaluated in exploratory subgroup analyses nor the competing risk of death sensitivity analysis).

## Discussion

In this study, we set out to evaluate the risk of subsequent fractures in subjects with opportunistically identified VF on routine CT scans. First, as a group, participants undergoing routine CT scans were a high‐risk population in terms of fracture risk relative to the general population. Second, within this group, patients having identifiable VF on the CT scan—and not treated for osteoporosis—were at additionally increased risk of future MOF, in particular hip fracture. Despite of these observations, we noted that only 9% of subjects with VF were censored due to initiation of OM therapy.

While two studies have reported the risk of fractures in subjects with opportunistically identified VF,^(^ ^14^, ^25^ ^)^ this is the first study to evaluate the *relative* risk of *any* subsequent fracture as well as non‐hip fractures in subjects with opportunistically identified VF on routine CT scans, to compare these risks against a general population sample, and to fully discount the effect of treatment with OMs when assessing these risks. Despite of this novelty, the point estimate of the primary outcome (risk of any subsequent fracture) was lower than expected given the fracture risk associated with clinical and screening‐detected VF, as reported from other studies. For example, in the Dubbo Osteoporosis Epidemiology Study, relative risks of subsequent fractures after a clinical VF were 2.52 and 6.18 in women and men, respectively.^(^ ^22^ ^)^ In the Study of Osteoporotic Fractures, the relative risk of any nonvertebral fracture was 1.5 in women with versus women without prevalent VF at baseline identified systematically by thorax and lumbar radiographs.^(^ ^24^ ^)^ Although study populations and VF definitions in these studies differ from ours, the observed risk estimates are somewhat contrasting. There are important aspects to consider: First, the sensitivity analysis introducing a competing risk of death showed that the point estimate was reduced to below 1, indicating a high competing risk of death in the VF cohort, which in the statistical analyses would be protective against subsequent fractures. Second, the subgroup analysis stratifying the primary outcome according to the worst VF at baseline showed that the HR point estimate in subjects with mild VF was close to 1, indicating that these fractures did not appear to confer an increase in the risk of any subsequent fractures, and their inclusion would have reduced the overall HR toward 1. Third, it is important to note that the incidence rate (IR) of any fracture was high in both the VF cohort (IR 41.52 fractures per 1000 subject‐years) and the no VF cohort (IR 31.38), if compared to the general population cohort (IR 21.80). Again, this emphasizes that the CT scanned population in itself was a high‐risk group for fractures. Of note, the general population cohort estimate is, reassuringly, similar to a previously reported estimate of the incidence of any fracture in men and women aged 50 years or older living in Denmark (IR 249 per 10,000 subject‐years).^(^ ^33^ ^)^

The increase in the risk of hip fracture and subsequent VF (relative risk of VF not significant within the CT population) identified in this study are important, given the poor outcomes associated with these fracture types.^(^ ^36^, ^37^, ^38^ ^)^ The hip fracture risk (HR_adj_ 3.02) was similar to the risk of future hip fractures in subjects with VF identified on routine chest CT scans (HR 3.1 [95% CI, 2.1–4.7]), as reported by Buckens and colleagues.^(^ ^25^ ^)^ Comparing subjects with and without VF on CT pulmonary angiograms, Jones and colleagues^(^ ^14^ ^)^ reported a higher cumulative incidence of hip fracture (11.1% versus 2.8%; *p* = 0.03) during up to 4.5 years of follow‐up. These findings in subjects with opportunistically identified VF are congruent with the hip fracture risk reported for women with systematically identified VF on baseline radiographs.^(^ ^21^, ^24^ ^)^

The high risk of MOF, driven by hip and VF, in subjects with opportunistically identified VF certainly merits further evaluation for osteoporosis and treatment with an OM if appropriate. Using the prevalence of subjects with VF not excluded from the study population (*n* = 332/2000) and the cumulative incidence of MOF within this patient group (*n* = 26/332), and assuming full uptake of OM in VF subjects and a relative risk reduction for fractures by OM therapy of 50%, the number needed to screen^(^ ^39^ ^)^ to prevent one MOF during up to 7 years of follow‐up is 154. To put this into perspective, this estimate is substantially lower than for any of the seven strategies to prevent cardiovascular disease evaluated by Chamnan and colleagues,^(^ ^40^ ^)^ with the caveat of different modeling assumptions between the studies. Combined with the fact that as little as 9% of the patients in our study went on to receive treatment for osteoporosis during the 7 years following the index CT scan, screening and sufficient reporting—a prerequisite to allow further evaluation—of VFs available on CT scans would likely represent an important added value to a substantial number of patients. Publicly available data (www.esundhed.dk) report that the total number of CT scans including the chest and/or abdomen amounted to approximately 645,000–751,000 (variation depending on the categories of CT scans included in the count) in Denmark in 2021. Clearly this will amount to a somewhat lower number of unique persons, due to the need for repeat exams or diagnostic workup for more than one medical condition in the same year in some patients. If we assume that the average number of CT exams is as high as four scans per individual, these scans would have been performed in 161,250 to 187,750 unique individuals. We consider this a conservative estimate of the number of subjects who could be covered by opportunistic screening for VFs in Denmark per calendar year. Based on the number needed to screen calculated above, systematic screening for VF for 1 year could save 1047 to 1219 MOFs in Denmark over the subsequent 7 years.

There are limitations to this study. First, there is—as described previously—a risk of nondifferential misclassification of exposure. This could occur if subjects in the no VF (comparator) cohort did in fact have a VF outside the CT field of view—and as such would be erroneously classified as unfractured. In the sensitivity analysis for the risk of any subsequent fracture (primary outcome) excluding matched pairs where the comparator had only a subset of the thoracic and lumbar spine visible on the CT, a substantially higher HR (HR_adj_ 1.81) was identified, increasing the plausibility of this bias being present, which would drive the relative risk estimate toward 1. Second, a limitation lies in the use of register data, with an inevitable risk of primary coding errors. This would, however, not be expected to cause any systematic bias. Third, as compared to the assumptions set out in the sample size calculation, the size of the analysis population was smaller and the subsequent fracture rate was lower. Although this reduced the power of the study, statistically significant differences were still observed for the most serious clinical outcomes (MOF and hip fracture). Finally, we did not have access to the radiological reports from the CT scans, and thus were unable to ascertain if the VF had been reported or not. Importantly, this was not pertinent to our study purpose, evaluating fracture risk in subjects with VFs not treated with OMs.

The strengths of this study include the large study population and the potential for up to 7 years follow‐up. Furthermore, the identification of VFs in a two‐tiered process blinded to clinical information ensured the validity of the VF classification. Finally, the design of this study using a real‐world patient population, ensures that the findings are directly applicable to clinical practice.

In conclusion, this study demonstrated that subjects with opportunistically identified VF on routine CT scans are at highly increased risk of MOF and hip fracture. This was observed both when compared to subjects with no VF on the CT scan (HR_adj_ 1.72 and 3.02, respectively) as well as to the general population (HR_adj_ 2.04 and 2.55, respectively). We assessed this by excluding patients who had received therapy with an OM in the past year before the CT scan and by censoring subjects at the point of initiation of any OM after the date of the scan. The risk estimates therefore accurately reflects the risk seen in patients who are not treated with OMs. Indeed, absence of treatment was noted in the great majority of VF cases, with only 9% of subjects with VF being censored from this study due to initiation of an OM, suggesting a considerable treatment gap. Together, these findings add to the evidence base supporting systematic identification of subjects with VF available on routine CT scans, to enable further fracture risk evaluation and management, effectively increasing the individual and societal utility of the radiation and costs associated with CT scans.

**Table:** 

What is already known on the topic VFs are one of the most common fragility fractures in the aging population, and cohort studies have found that they are associated with future fractures and death. VFs are a common chance finding on routine radiological imaging, yet auditing studies have found that they are often either missed or ambiguously reported. Randomized controlled trials (RCTs) have demonstrated that patients with osteoporotic VFs benefit from inexpensive bone‐protective medications.
What does this study add? Patients undergoing CT scans—and not treated for osteoporosis—constitute a high risk population for subsequent fractures. Within the CT population—and when compared to the general population—patients with opportunistically identified VF are at strongly elevated risk of major osteoporotic fracture, especially hip fracture, in the absence of osteoporosis treatment.

## Author Contributions

MKS: Conceptualization, data curation, formal analysis, funding acquisition, investigation, methodology, project administration, writing original draft, review & editing of manuscript. JN: Data curation, review & editing of manuscript. CDS: Data curation, formal analysis, review & editing of manuscript. KRO: Methodology, review & editing of manuscript. CC: Methodology, review & editing of manuscript. CL: Conceptualization, data curation, methodology, review & editing of manuscript. BA: Conceptualization, funding acquisition, methodology, supervision, review & editing of manuscript. All authors had full access to the study results, and assumes responsibility for the decision to submit for publication. Guarantors: MKS and BA. The guarantors affirm that this manuscript is an honest, accurate, and transparent account of the study being reported; that no important aspects of the study have been omitted; and that any discrepancies from the study as originally planned have been explained. The corresponding author attests that all listed authors meet authorship criteria and that no others meeting the criteria have been omitted.

## Disclosures

All authors have completed the ICMJE uniform disclosure form at http://www.icmje.org/disclosure-of-interest/ and declare: MKS and BA have received support for this study from UCB/Amgen and Region Zealand Health Scientific Research Foundation (research grants with funds paid to the institution); MKS received support from the University of Southern Denmark (PhD scholarship), UCB (educational grant) outside the submitted work, is a board member of the Danish Bone Society, and a member of working groups in the Danish Bone Society and the European Calcified Tissue Society; JN and CL are employees of UCB Pharma with stock ownership, and JN is involved in a patent (WO2019/106061) and has received support for travel from UCB Pharma; CDS and KRO have no conflicts to report; CC has received personal fees from Alliance for Better Bone Health, Amgen, Eli Lilly, GSK, Medtronic, Merck, Novartis, Pfizer, Roche, Servier, Takeda, and UCB; BA has received personal speakers fees/consulting fees from UCB, Amgen, Kyowa‐Kirin, and Pharmacosmos, and institutional research grants (with funds paid to the institution) from Novartis, Kyowa‐Kirin, and Pharmacosmos, and is the president of the European Calcified Tissue Society.

### Peer Review

The peer review history for this article is available at https://publons.com/publon/10.1002/jbm4.10736.

## Supporting information


**Data S1.** Supporting Information.
Tables S1–S6.
Figs. S1–S3.

## Data Availability

MKS and CDS had full access to individual‐level data of all subjects in this study and performed the data analysis. Under Danish law sharing of these individual‐level data is not possible.
