# Supplementary material for: Fracture Risk in Men and Women With Vertebral Fractures Identified Opportunistically on Routine Computed Tomography Scans and Not Treated for Osteoporosis: An Observational Cohort Study
Source: JBMR Plus. 2023 Mar 15;7(5):e10736. doi: 10.1002/jbm4.10736 (PMC10184021; doi:10.1002/jbm4.10736)
Supplement: Supplementary file 1 — Data S1. Supporting Information. Tables S1–S6. Figs. S1–S3. [file JBM4-7-e10736-s001.docx]

**Fracture risk in men and women with vertebral fractures identified opportunistically on routine CT scans and not treated for osteoporosis: An observational cohort study**

**Supplementary appendix**

Michael Kriegbaum Skjødt, MD GDBA^1,2^, Joeri Nicolaes, MSc^3,4^, Christopher Dyer Smith, PhD^2^, Kim Rose Olsen, PhD^5^, Cyrus Cooper, MD PhD^6,7^, Cesar Libanati, MD^3^, Bo Abrahamsen, MD PhD^1,2,7^

^1^Department of Medicine, Holbæk Hospital, Holbæk, Denmark

^2^OPEN – Open Patient data Explorative Network, Department of Clinical Research, University of Southern Denmark and Odense University Hospital, Odense, Denmark

^3^UCB Pharma, Brussels, Belgium

^4^Medical Image Computing, ESAT-PSI, Department of Electrical Engineering, KU Leuven, Leuven, Belgium

^5^DaCHE, Institute of Public Health, University of Southern Denmark, Odense, Denmark

^6^MRC Lifecourse Epidemiology Center, University of Southampton, Southampton General Hospital, Southampton, UK

^7^NDORMS, Nuffield Department of Orthopaedics, Rheumatology and Musculoskeletal Sciences, Oxford University Hospitals, Oxford, UK

**Corresponding author**

Michael Kriegbaum Skjødt, MD GDBA (ORCiD 0000-0002-2109-8989)

Department of Medicine, Holbæk Hospital, Smedelundsgade 60, DK-4300 Holbæk, Denmark

Email: [miksk@regionsjaelland.dk](mailto:miksk@regionsjaelland.dk)

Tel.: 0045 61676764

**Supplemental Table 1: Variable definitions**

| **Variable ID** | **Description** | **Register** | | |
| --- | --- | --- | --- | --- |
|  |  | **National Patient Register** | | **Register of Medicinal Product Characteristics**  ATC-codes |
|  |  | **Diagnosis codes**  ICD-10 codes | **Operation/procedure codes**  SKS-codes |  |
| **1** | **Major osteoporotic fractures** |  |  |  |
| 1.1 | Fracture of hip^ⴕ^ | S72.0, S72.1, S72.2 | KNFB.0-99, KNFJ.4-9 |  |
| 1.2 | Fracture of vertebra (spine) |  |  |  |
| 1.2.1 | Fracture of thoracic vertebra (spine) | S22.0, S22.1 |  |  |
| 1.2.2 | Fracture of lumbar vertebra (spine) | S32.0, S32.7 (not S32.7B), S32.8A |  |  |
| 1.2.3 | Fracture of vertebra (spine), unspecified level | T08, T08.9 |  |  |
| 1.3 | Fracture of humerus | S42.2, S42.3 |  |  |
| 1.4 | Fracture of distal forearm | S52.5, S52.6, S52.8C |  |  |
| **2** | **Other (non-major) fractures** |  |  |  |
| 2.1 | Fracture of cervical vertebra (spine) | S12.0, S12.1, S12.2, S12.7, S12.9 |  |  |
| 2.2 | Fracture of clavicle or scapula | S42.0, S42.1, S42.7A, S42.7C |  |  |
| 2.3 | Fracture of femur (non-hip) | S72, S72.3, S72.4, S72.7, S72.8, S72.9 |  |  |
| 2.4 | Fracture of forearm (proximal and other) | S52, S52.0, S52.1, S52.2, S52.3, S52.4, S52.7, S52.8 (not S52.8C), S52.9 |  |  |
| 2.5 | Fracture of foot and toes | S92, S92.x |  |  |
| 2.6 | Fracture of humerus (other) | S42, S42.4, S42.7 (not S42.7A or C), S42.8, S42.9 |  |  |
| 2.7 | Fracture of lower leg, incl. knee and ankle | S82, S82.x |  |  |
| 2.8 | Fractures of multiple regions | T02.x |  |  |
| 2.9 | Fracture of os sacrum | S32.1, S32.2 |  |  |
| 2.10 | Fracture of pelvis | S32.3, S32.4, S32.5, S32.7B, S32.8 (not S32.8A) |  |  |
| 2.11 | Fracture of thorax (sternum, rib, and unspecified) | S22, S22.2, S22.3, S22.4, S22.8, S22.9 |  |  |
| 2.12 | Fracture of upper and lower limb, unspecified  and unspecified body region | T10, T10.9, T12, T12.9, T14.2 |  |  |
| 2.13 | Fracture of wrist | S62, S62.0, S62.1, S62.8 |  |  |
| **3** | **Fracture of face, skull, or fingers** | S02, S02.0, S02.1, S02.2, S02.3, S02.4, S02.6, S02.7, S02.8, S02.9, S62.2, S62.3, S62.4, S62.5, S62.6, S62.7 |  |  |
| **4** | **Risk factors for osteoporosis/fractures** |  |  |  |
| 4.1 | Osteoporosis | M80.x, M81.x, M82.x |  |  |
| 4.2 | Hyperthyroidism | E05.x |  | H03B |
| 4.3 | Primary hyperparathyroidism | E21.0 |  |  |
| 4.4 | Cushings syndrome | E24.x |  |  |
| 4.5 | Hypogonadism (male) | E23.0A, E89.5, E29.1 |  | G03BA03 |
| 4.6 | Acromegaly | E22.0 |  |  |
| 4.7 | Addisons disease | E27.1 (not E27.1B) |  |  |
| 4.8 | Monogenetic osteoporosis (osteogenesis  imperfecta, Ehlers-Danlos syndrome, Marfans  syndrome) | Q78.0, Q79.6, Q87.4 |  |  |
| 4.9 | Anorexia nervosa | F50.0, F50.1 |  |  |
| 4.10 | Bulimia | F50.2, F50.3 |  |  |
| 4.11 | Malabsorption |  |  |  |
|  | Coeliac disease | K90.0 |  |  |
|  | Postgastrectomy | K91.1, K91.2 (not K91.2B/C) |  |  |
|  | Short bowel syndrome | K91.2B |  |  |
|  | Pancreas insufficiency (chronic pancreatitis) | K86.0, K86.1 |  |  |
| 4.12 | Inflammatory bowel disease (Crohns disease,  ulcerative colitis) | K50.x, K51.x |  |  |
| 4.13 | Pernicious anaemia | D51.0 |  |  |
| 4.14 | Spondylitis anchylopoietica/Bechterews disease | M45.x |  |  |
| 4.15 | Mastocytosis | Q82.2 |  |  |
| 4.16 | Systemic lupus erythematosus | M32.x |  |  |
| 4.17 | Juvenile arthritis | M08.x (not M08.0), M09.x |  |  |
| 4.18 | Rheumatoid arthritis | M05*, M05.0, M05.1, M05.3, M05.8, M05.9, M06.0, M06.1, M06.8, M06.9, M08.0 |  |  |
| 4.19 | Myelomatosis | C90.0 |  |  |
| 4.20 | Type 1 diabetes mellitus | E10.x |  |  |
| 4.21 | Type 2 diabetes mellitus | E11.x |  |  |
| 4.22 | Paget’s disease of bone | M88.x |  |  |
| 4.23 | Cancer |  |  |  |
| 4.23.1 | Malignancies | C00.x–C26.x, C30.x–C34.x, C37.x–C41.x, C43.x–C58.x, C60.x–C86.x, C88.x, C90.x–C97.x |  |  |
| 4.23.2 | Benign tumors | D10.x, D11.x, D12.x, D13.x, D14.x, D15.x, D16.x, D17.x, D18.x, D19.x, D20.x, D21.x, D22.x, D23.x, D24, D25.x, D26.x, D27, D28.x, D29.x, D30.x, D31.x, D32.x, D33.x, D34, D35.x, D36.x |  |  |
| 4.23.3 | Malignancies or benign tumors (unknown) | D37.x, D38.x, D39, D40, D41, D42, D43, D44, D45, D46, D47, D48 |  |  |
| **5** | **Medications** |  |  |  |
| 5.1 | Anti-osteoporosis medication |  |  |  |
| 5.1.1 | Selective estrogen receptor modulators  (raloxifene) |  |  | G03XC01 |
| 5.1.2 | Bisphosphonates |  |  |  |
| 5.1.2.1 | Etidronic acid |  |  | M05BA01, M05BB01 |
| 5.1.2.2 | Clodronic acid |  |  | M05BA02, M05BB02 |
| 5.1.2.3 | Pamidronic acid |  | MM05BA03 | M05BA03 |
| 5.1.2.4 | Alendronic acid |  |  | M05BA04, M05BB03, M05BB05, M05BB06 |
| 5.1.2.5 | Tiludronic acid |  |  | M05BA05 |
| 5.1.2.6 | Ibandronic acid |  | MM05BA06, MM05BB09 | M05BA06, M05BB09 |
| 5.1.2.7 | Risedronic acid |  |  | M05BA07, M05BB04, M05BB07 |
| 5.1.2.8 | Zoledronic acid (or any hospital-administered  bisphosphonate for procedure codes) |  | BWHB40, MM05BA08, MM05BB08 | M05BA08, M05BB08 |
| 5.1.3 | Strontium ranelate |  |  | M05BX03, M05BX53 |
| 5.1.4 | Denosumab |  | BWHB42, MM05BX04 | M05BX04 |
| 5.1.5 | Teriparatide |  |  | H05AA02 |
| 5.2 | Opposed HRT (progestogens and estrogens in  combination) |  |  | G03F, G03C |
| 5.3 | Antidiabetic drugs |  |  |  |
| 5.3.1 | Insulin agents |  |  | A10A |
| 5.3.2 | Non-insulin agents |  |  | A10B |
| 5.3.2.1 | SGLT-2 inhibitors |  |  | A10BK |
| 5.3.2.2 | Thiazolidinediones |  |  | A10BG |
| **6** | **Medications associated with an increased risk of osteoporosis/fractures** |  |  |  |
| 6.1 | Glucocorticoids |  |  |  |
|  | Cortisone |  |  | H02AB10 |
|  | Hydrocortisone |  |  | H02AB09 |
|  | Methylprednisolone |  |  | H02AB04 |
|  | Prednisolone/prednisone |  |  | H02AB06, H02AB07 |
|  | Triamcinolone |  |  | H02AB08 |
|  | Betamethasone |  |  | H02AB01 |
|  | Dexamethasone |  |  | H02AB02 |
| 6.2 | Anticoagulants (vitamin K antagonists, heparin) |  |  | B01AA, B01AB |
| 6.3 | Antidepressants (selective serotonine reuptake  inhibitors, venlafaxine, duloxetine) |  |  | N06AB, N06AX16, N06AX21 |
| 6.4 | Cancer agents (aromatase inhibitors, calcineurin  inhibitors, cyclophosphamide, methotrexate) |  |  | L02BG, L04AD, L01AA01, L01BA01, L04AX03 |
| 6.5 | Antiepileptics (valproic acid, carbamazepine,  phenobarbital, phenytoin) |  |  | N03AG01, N03AF01, N03AA02, N03AB02, N03AB52 |
| 6.6 | GnRH agonists |  |  | L02AE |
| 6.7 | Proton pump inhibitors |  |  | A02BC |
| **7** | **Charlson comorbidity index (CCI)** |  |  |  |
| 7.1 | Myocardial infarction | I21.x, I22.x, I25.2 |  |  |
| 7.2 | Congestive heart failure | I09.9, I11.0, I13.0, I13.2, I25.5, I42.0, I42.5-I42.9, I43.x, I50.x, P29.0 |  |  |
| 7.3 | Peripheral vascular disease | I70.x, I71.x, I73.1, I73.8, I73.9, I77.1, I79.0, I79.2, K55.1, K55.8, K55.9, Z95.8, Z95.9 |  |  |
| 7.4 | Cerebrovascular disease | G45.x, G46.x, H34.0, I60.x–I69.x |  |  |
| 7.5 | Dementia | F00.x–F03.x, F05.1, G30.x, G31.1 |  | N06DA02, N06DA03, N06DA04, N06DX01 |
| 7.6 | Chronic pulmonary disease | I27.8, I27.9, J40.x–J47.x, J60.x–J67.x, J68.4, J70.1, J70.3 |  |  |
| 7.7 | Rheumatic disease | M05.x, M06.x, M31.5, M32.x–M34.x, M35.1, M35.3, M36.0 |  |  |
| 7.8 | Peptic ulcer disease | K25.x–K28.x |  |  |
| 7.9 | Mild liver disease | B18.x, K70.0–K70.3, K70.9, K71.3–K71.5, K71.7, K73.x, K74.x, K76.0, K76.2–K76.4, K76.8, K76.9, Z94.4 |  |  |
| 7.10 | Diabetes without chronic complication | E10.0, E10.1, E10.6, E10.8, E10.9, E11.0, E11.1, E11.6, E11.8, E11.9, E12.0, E12.1, E12.6, E12.8, E12.9, E13.0, E13.1, E13.6, E13.8, E13.9, E14.0, E14.1, E14.6, E14.8, E14.9 |  | A10A, A10B^Φ^ |
| 7.11 | Diabetes with chronic complication | E10.2–E10.5, E10.7, E11.2–E11.5, E11.7, E12.2–E12.5, E12.7, E13.2–E13.5, E13.7, E14.2–E14.5, E14.7 |  |  |
| 7.12 | Hemiplegia or paraplegia | G04.1, G11.4, G80.1, G80.2, G81.x, G82.x, G83.0–G83.4, G83.9 |  |  |
| 7.13 | Renal disease | I12.0, I13.1, N03.2–N03.7, N05.2–N05.7, N18.x, N19.x, N25.0, Z49.0–Z49.2, Z94.0, Z99.2 |  |  |
| 7.14 | Any malignancy, including lymphoma and  leukemia, except malignant neoplasm of skin | C00.x–C26.x, C30.x–C34.x, C37.x–C41.x, C43.x, C45.x–C58.x, C60.x–C76.x, C81.x–C85.x, C88.x, C90.x–C97.x |  |  |
| 7.15 | Moderate or severe liver disease | I85.0, I85.9, I86.4, I98.2, K70.4, K71.1, K72.1, K72.9, K76.5, K76.6, K76.7 |  |  |
| 7.16 | Metastatic solid tumor | C77.x–C80.x |  |  |
| 7.17 | HIV/AIDS | B20.x–B22.x, B24.x |  |  |

**Supplemental Table 1: Variable definitions**
Each covariate is considered present upon occurrence of a relevant diagnosis code, operation/procedure code, and/or ATC code. For baseline characteristics (baseline is defined as the date of the CT scan or – for the general population comparator cohort – the date of the CT scan of the matched case), any occurrence prior to baseline fulfills the criteria for the variable being present at baseline.
ATC, Anatomical Therapeutic Chemical Classification System; CCI, Charlson Comorbidity Index; GnRH, Gonadotropin Releasing Hormone; HIV/AIDS, Human Immunodeficiency Virus/Acquired Immunodeficiency Syndrome; HRT, hormone replacement therapy; ICD-10, International Classification of Diseases, 10^th^ edition; SGLT2-inhibitor, sodium-glucose cotransporter-2 inhibitor; SKS, Sundhedsvæsenets Klassifikations System (a Danish coding system).
^ⴕ^For hip fractures, more granular criteria for occurrence exist: Hospital admission with a primary diagnosis code for hip fracture and within that admission a procedure-code for hip surgery including specification of affected side. Surgery codes are available under the applied nomenclature since 1996, hence hip fractures occurring earlier than 1996 are not included.
*Added post hoc.
^Φ^Biguanides (ATC-code A10BA) added post hoc.

**Supplemental Table 2: Accident codes**

| **Description** | **National Patient Register** |
| --- | --- |
| Accident codes | EUP2, EUP3, EUP4, EUP5, EUP7, EUM3, EUM4, EUM5, EUM7, EUM8, EUBB |

**Supplemental Table 2: Accident codes**This table shows the accident codes associated with high-energy trauma.

**Supplemental Table 3: Subgroup analyses for the primary outcome**

|  | **Definition of subgroup analyses** | **Unadjusted (crude)** | | **Adjusted** | |
| --- | --- | --- | --- | --- | --- |
|  |  | HR (95% CI) | P_interaction_ | HR (95% CI) | P_interaction_ |
| **Primary outcome** |  | **1.30 (0.85-2.00)** |  | **1.31 (0.85-2.03)** |  |
| **Subgroup analyses** |  |  |  |  |  |
| Age group at baseline  50-59 years (n=81)  60-69 years (n=283)  70-79 years (n=336)  80+ years (n=227) | Age at baseline. | 0.42 (0.09-1.93)  1.07 (0.50-2.30)  2.31 (1.09-4.89)*  1.63 (0.64-4.17) | N/A | 0.18 (0.03-0.95)*  1.07 (0.49-2.35)  2.68 (1.24-5.80)*  1.46 (0.58-3.73) | N/A |
| Sex  Female (n=433)  Male (n=494) | Sex. | 1.45 (0.83-2.53)  1.15 (0.58-2.28) | N/A | 1.50 (0.85-2.65)  1.07 (0.53-2.13) | N/A |
| Malignancies within 3 years before baseline  No (n=648)  Yes (n=279) | ≥1 diagnosis code for any malignancy within 3 years before baseline. | 1.34 (0.82-2.17)  1.17 (0.46-2.98) | 0.48 | 1.34 (0.82-2.19)  1.20 (0.47-3.07) | 0.48 |
| Bone diseases at baseline | ≥1 diagnosis code for Paget’s disease (M88), osteogenesis imperfecta (Q78.0), primary hyperparathyroidism (E21.0), myelomatosis (C90), Marfans disease (Q87.4)^ⴕ^ and/or Ehlers-Danlos syndrome (Q79.6)^ⴕ^ at any time prior to baseline. | Not reportable due to n<5 in one or more subgroups. | | | |
| Osteoporosis at baseline  No (n=890)  Yes (n=37) | ≥1 diagnosis code for osteoporosis and/or ≥1 treatment with OM at any time prior to baseline. | 1.16 (0.74-1.84)  3.03 (0.35-26.17) | 0.49 | Results not interpretable |  |
| Number of VF  1 (n=415)  2 (n=233)  3 (n=111)  4+ (n=168) | Number of VF at baseline of the case in each matching group, according to the reference standard reading. | 1.78 (0.92-3.43)  1.25 (0.55-2.85)  0.77 (0.24-2.40)  1.12 (0.36-3.49) | 0.48 | 1.74 (0.89-3.40)  1.48 (0.65-3.40)  0.81 (0.26-2.54)  1.00 (0.32-3.14) | 0.46 |
| Worst VF  Mild (n=286)  Moderate (n=339)  Severe (n=302) | Worst VF at baseline of the case in each matching group, according to the reference standard reading. | 0.92 (0.38-2.22)  1.14 (0.57-2.28)  2.15 (1.06-4.37)* | 0.20 | 0.94 (0.39-2.27)  1.14 (0.57-2.31)  2.08 (1.01-4.27)* | 0.25 |
| Position of VF  Thorax (n=468)  Lumbar (n=194)  Both thorax and lumbar   (n=265) | Position of VF at baseline of the case in each matching group, according to the reference standard reading. | 1.25 (0.68-2.27)  1.67 (0.62-4.45)  1.20 (0.55-2.63) | 0.63 | 1.29 (0.70-2.36)  1.79 (0.66-4.80)  1.09 (0.49-2.42) | 0.57 |
| CCI score at baseline ^ⴕ^  0 (n=358)  1 (n=102)  2 (n=246)  3+ (n=221) | Charlson comorbidity index score at baseline. | 1.39 (0.69-2.79)  3.66 (1.05-12.74)*  0.74 (0.33-1.70)  1.05 (0.42-2.65) | 0.24 | 1.47 (0.72-2.97)  3.07 (0.88-10.72)  0.75 (0.32-1.72)  1.08 (0.43-2.71) | 0.32 |

**Supplemental Table 3: Subgroup analyses for the primary outcome**This table shows the definition and results – crude and adjusted, respectively – of the subgroup analyses for the primary outcome. The primary outcome is the risk of any subsequent fracture - except face, skull, and fingers – after baseline in the VF on CT scan (exposed) cohort versus the no VF on CT scan (comparator) cohort. In the adjusted models matching has been lifted, and the confounders included are similar to those identified for the primary outcome: Age, sex (except in the subgroup analyses based on sex), baseline presence of any prior fracture, anorexia, ever use of antidepressants, and ever use of proton pump inhibitors. A p-value for interaction is not available for the subgroup analyses on age and sex (the variables used for matching), as these were conducted by running independent analyses for each subgroup.
CCI, Charlson Comorbidity Index; CI, confidence interval; CT, computed tomography; HR, hazard ratio; OM, osteoporosis medication; VF, vertebral fracture.
^ⴕ^Added during the analysis phase (i.e. post hoc).
*p<0.05.

**Supplemental Table 4: Sensitivity analyses for the primary outcome**

|  | **Definition of sensitivity analyses** | **Unadjusted (crude)** | **Adjusted** |
| --- | --- | --- | --- |
|  |  | HR (95% CI; p-value) | HR (95% CI; p-value) |
| **Primary outcome** |  | **1.30 (0.85-2.00; p=0.23)** | **1.31 (0.85-2.03; p=0.23)** |
| **Sensitivity analyses** |  |  |  |
| Loss to follow-up | Count subjects migrating out of Denmark (missing) as fractured at the date of migration. | 1.28 (0.83-1.97; p=0.26) | 1.29 (0.84-2.00; p=0.25) |
| Exclude if OM at any time before baseline | Exclude subjects treated with OM at any time prior to baseline. | 1.13 (0.71-1.79; p=0.61) | 1.14 (0.72-1.83; p=0.57) |
| Censor if ≥2 treatments with osteoporosis medications | Censor at the time of first treatment with OM after baseline if the subject receives at least 2 treatments for osteoporosis after baseline (filled prescriptions for and/or hospital administration of OM). | 1.28 (0.83-1.96; p=0.26) | 1.28 (0.83-1.98; p=0.26) |
| Misclassification bias 1: Exclude comparators with prior diagnosis of VF | Exclusion of subjects in the comparator cohort with a diagnosis code for thoracic, lumbar, and/or any VF (Supplemental Table 1, Variable IDs 1.2.1-1.2.3) prior to baseline, to reduce the risk of misclassification of exposure. | 1.30 (0.84-1.99; p=0.24) | 1.30 (0.84-2.01; p=0.24) |
| Misclassification bias 2: Exclude comparators without full T1-L5 visible on CT scan^ⴕ^ | Exclusion of subjects in the comparator cohort if one or more thoracic or lumbar vertebrae are not available on the index CT - i.e. all vertebrae from T1 to L5 must be visible - to reduce the risk of misclassification of exposure. | 1.85 (0.90-3.82; p=0.09) | 1.81 (0.86-3.81; p=0.12) |
| Competing risk of death^ⴕ^ | Evaluation of the primary outcome using a model taking the competing risk of death into account. The analysis was performed using the stcrreg command (Stata 16/17), treating death as a competing risk. Matching has been lifted for the crude analysis, and instead sex and age have been added to the model. | 0.94 (0.61-1.45; p=0.79) | 0.87 (0.56-1.36; p=0.55) |
| Residual confounding | Evaluation of the potential for residual confounding by a placebo-analysis using acute appendicitis (diagnosis codes K35-37) as the pseudo-outcome. | 0.58 (0.06-5.15; p=0.62) | 0.47 (0.05-4.33; p=0.51) |

**Supplemental Table 4: Sensitivity analyses for the primary outcome**This table shows the definition and results – crude and adjusted, respectively – of the sensitivity analyses for the primary outcome. The primary outcome is the risk of any subsequent fracture - except face, skull, and fingers – after baseline in the VF on CT scan (exposed) cohort versus the no VF on CT scan (comparator) cohort. In the adjusted models matching has been lifted, and the confounders included are similar to those identified for the primary outcome: Age, sex, baseline presence of any prior fracture, anorexia, ever use of antidepressants, and ever use of proton pump inhibitors.
BL, baseline; CI, confidence interval; HR, hazard ratio; OM, osteoporosis medication; VF, vertebral fracture.
^ⴕ^Added during the analysis phase (i.e. post hoc).

**Supplemental Table 5: Scaling analysis population baseline characteristics**

|  | **VF on CT scan**  (exposed cohort) | **General population**  (comparator cohort) | **p-value** |
| --- | --- | --- | --- |
| N | 332 | 996 |  |
| Age, years; median (IQR) | 73 (65-79) | 73 (65-80) | 0.80 |
| Sex, men; n (%) | 181 (54.5%) | 543 (54.5%) | 1.00 |
| Country of origin, Denmark; n (%) | 323 (97.3%) | 967 (97.1%) | 0.95 |
| CCI-score; median (IQR) | 2 (0-3) | 0 (0-1) | <0.001 |
| Any prior fracture^1^; n (%) | 115 (34.6%) | 208 (20.9%) | <0.001 |
| Prior MOF; n (%) | 72 (21.7%) | 116 (11.6%) | <0.001 |
| Prior VF^2^; n (%) | 21 (6.3%) | 10 (1.0%) | <0.001 |
| Osteoporosis; n (%) | 15 (4.5%) | 18 (1.8%) | 0.006 |
| Glucocorticoid therapy^3^; n (%) | 55 (16.6%) | 66 (6.6%) | <0.001 |
| Hormone replacement therapy^3^; n (%) | 15 (4.5%) | 73 (7.3%) | 0.07 |
| Malignancies; n (%) | 132 (39.8%) | 124 (12.4%) | <0.001 |
| Rheumatoid arthritis; n (%) | 7 (2.1%) | 14 (1.4%) | 0.37 |
| Type 1 diabetes mellitus; n (%) | 18 (5.4%) | 22 (2.2%) | 0.003 |
| Type 2 diabetes mellitus; n (%) | 40 (12.0%) | 59 (5.9%) | <0.001 |

**Supplemental Table 5: Scaling analysis population baseline characteristics**
CCI, Charlson Comorbidity Index; CT, computed tomography; IQR, interquartile range; MOF, major osteoporotic fracture; N/A, not available; VF, vertebral fracture.
^1^Not including face, skull, or fingers. ^2^Including cervical VF. ^3^In the year prior to baseline.

**Supplemental Table 6: Risk of subsequent fractures for the VF on CT scan (exposed) cohort vs the general population (comparator) cohort – scaling analysis**

| **Future fracture type** | **Fracture counts** Number (%) of subjects with first fracture | | **Incidence rate** per 1,000 subject-years (95% CI) | | **Risk estimate** Hazard ratio (95% CI; p-value) | |
| --- | --- | --- | --- | --- | --- | --- |
|  | **VF on CT scan**  (exposed cohort) | **General population** (comparator cohort) | **VF on CT scan**  (exposed cohort) | **General population** (comparator cohort) | **Crude** | **Adjusted^*^** |
| **Any** | 32 (9.6%) | 114 (11.4%) | 41.78  (29.54-59.08) | 21.80  (18.14-26.19) | 1.80  (1.21-2.68; p<0.01) | 1.60  (1.07-2.40; p=0.02) |
| **MOF** | 26 (7.8%) | 75 (7.5%) | 33.34  (22.70-48.97) | 14.19  (11.32-17.80) | 2.23  (1.41-3.53; p<0.001) | 2.04  (1.29-3.23; p<0.01) |
| **Other** | 14 (4.2%) | 53 (5.3%) | 17.76  (10.52-29.98) | 9.97  (7.62-13.05) | 1.68  (0.93-3.04; p=0.09) | 1.42  (0.78-2.61; p=0.25) |
| **Hip** | 14 (4.2%) | 36 (3.6%) | 17.60  (10.42-29.71) | 6.75  (4.87-9.36) | 2.52  (1.35-4.70; p<0.01) | 2.55  (1.35-4.81; p<0.01) |
| **Vertebral** | 6 (1.8%) | 7 (0.7%) | 7.43  (3.34-16.53) | 1.30  (0.62-2.73) | 5.91  (1.88-18.53; p<0.01) | 4.66  (1.51-14.36; p<0.01) |
| **Humerus^ⴕ^** | 18 (1.4%) | | 2.91  (1.84-4.63) | | 1.82  (0.58-5.74; p=0.31) | 1.73  (0.55-5.44; p=0.35) |
| **Distal forearm^ⴕ^** | 29 (2.2%) | | 4.72  (3.28-6.79) | | 0.92  (0.32-2.68; p=0.88) | 0.80  (0.27-2.35; p=0.69) |

**Supplemental Table 6: Risk of subsequent fractures for the VF on CT scan (exposed) cohort vs the general population (comparator) cohort – scaling analysis**The table demonstrates the fracture counts, incidence rates, and relative risk estimates for the risk of any subsequent fracture (except face, skull, and fingers) and according to subsequent fracture location in the scaling analysis (VF on CT scan [exposed] cohort vs the general population [comparator] cohort).
*Adjusted for sex, age, baseline presence of any prior fracture, myelomatosis, pernicious anaemia, primary hyperparathyroidism, diabetes mellitus type 1, and ever use of thiazolidinediones. Matching lifted for the adjusted analyses.
^ⴕ^Numbers pooled across the cohorts as number of events <5 in one of the cohorts.
CI, confidence interval; CT, computed tomography; MOF, major osteoporotic fracture; VF, vertebral fracture.

**Supplemental Figure 1: Log-log plot for the primary outcome (risk of any subsequent fracture)**

**
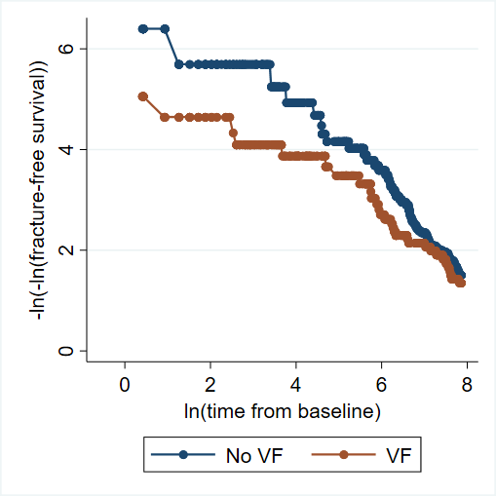
**

**Supplemental Figure 1: Log-log plot for the primary outcome (risk of any subsequent fracture)**
This figure demonstrates the log-log plot for the primary outcome (risk of any subsequent fracture – except face, skull, and fingers – in the VF on CT scan [exposed] cohort vs the no VF on CT scan [comparator] cohort).
CT, computed tomography; VF, vertebral fracture.

**Supplemental Figure 2: Cumulative probability of any subsequent fracture in the VF cohort vs the general population cohort**

**
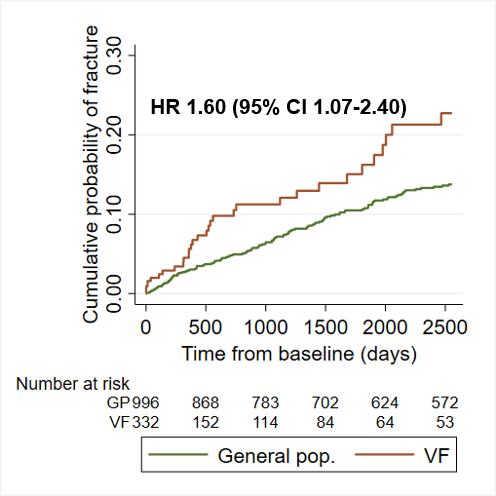
**

**Supplemental Figure 2: Cumulative probability of any subsequent fracture in the VF cohort vs the general population cohort**This Kaplan-Meier failure function demonstrates the cumulative probability of any subsequent fracture – except face, skull, and fingers – in the VF on CT scan (exposed) cohort vs the general population (comparator) cohort. Also shown is the hazard ratio for any subsequent fracture (except face, skull, and fingers), adjusted for age, sex, baseline presence of any prior fracture, myelomatosis, pernicious anaemia, primary hyperparathyroidism, diabetes mellitus type 1, and ever use of thiazolidinediones.
CI, confidence interval; CT, computed tomography; GP, general population; HR, hazard ratio; pop., population; VF, vertebral fracture.

**Supplemental Figure 3: Cumulative probability of fracture according to subsequent fracture location in the VF cohort vs the general population cohort**


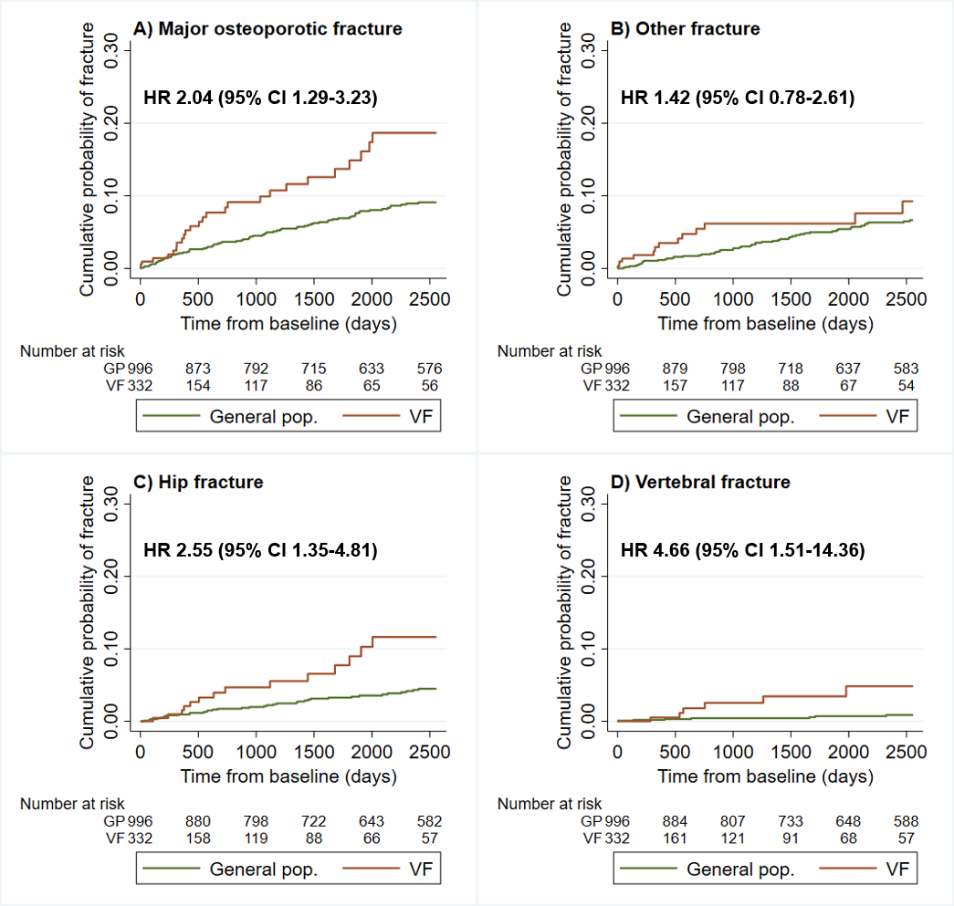


***Supplemental Figure 3: Cumulative probability of fracture according to subsequent fracture location in the VF cohort vs the general population cohort****Panel A, major osteoporotic fracture (MOF); B, other fracture; C, hip fracture; D, vertebral fracture.
The figure shows the Kaplan-Meier failure functions according to subsequent fracture location in the VF on CT scan (exposed) cohort vs the general population (comparator) cohort. On each panel is shown the hazard ratio (95% CI) for subsequent fracture according to subsequent fracture location, adjusted for age, sex, baseline presence of any prior fracture, myelomatosis, pernicious anaemia, primary hyperparathyroidism, diabetes mellitus type 1, and ever use of thiazolidinediones.
Panels for humerus fracture and distal forearm fracture not shown as number of events <5 in one of the cohorts.
CI, confidence interval; GP, general population; HR, hazard ratio; MOF, major osteoporotic fracture; pop., population; VF, vertebral fracture.*
